# Association of Six Single Nucleotide Polymorphisms with Gestational Diabetes Mellitus in a Chinese Population

**DOI:** 10.1371/journal.pone.0026953

**Published:** 2011-11-11

**Authors:** Ying Wang, Min Nie, Wei Li, Fan Ping, Yingying Hu, Liangkun Ma, Jinsong Gao, Juntao Liu

**Affiliations:** 1 Key laboratory of Endocrine, Ministry of Health, Department of Endocrinology, Peking Union Medical College Hospital, Peking Union Medical College, Chinese Academy of Medical Sciences, Beijing, China; 2 Department of Endocrinology, the Secondly Affiliated Hospital of ShanXi Medical College, Shan Xi, China; 3 Department of Obstetrics & Gynecology, Peking Union Medical College Hospital, Peking Union Medical College, Chinese Academy of Medical Sciences, Beijing China; Emory University School Of Medicine, United States of America

## Abstract

**Background:**

To investigate whether the candidate genes that confer susceptibility to type 2 diabetes mellitus are also correlated with gestational diabetes mellitus (GDM) in pregnant Chinese women.

**Methodology/Principal Findings:**

In this study, 1764 unrelated pregnant women were recruited, of which 725 women had GDM and 1039 served as controls. Six single nucleotide polymorphisms (rs7754840 in *CDKAL1*, rs391300 in *SRR*, rs2383208 in *CDKN2A/2B*, rs4402960 in IGF2BP2, rs10830963 in *MTNR1B*, rs4607517 in *GCK*) were genotyped using TaqMan allelic discrimination assays. The genotype and allele distributions of each SNP between the GDM cases and controls and the combined effects of alleles for the risk of developing GDM were analyzed. We found that the rs4402960, rs2383208 and rs391300 were statistically associated with GDM (OR = 1.207, 95%CI = 1.029–1.417, p = 0.021; OR = 1.242, 95%CI = 1.077–1.432, p = 0.003; OR = 1.202, 95%CI = 1.020–1.416, P = 0.028, respectively). In addition, the effect was greater under a recessive model in rs391300 (OR = 1.820, 95%CI = 1.226–2.701, p = 0.003). Meanwhile, the joint effect of these three loci indicated an additive effect of multiple alleles on the risk of developing GDM with an OR of 1.196 per allele (p = 1.08×10^−4^). We also found that the risk alleles of rs2383208 (b = −0.085, p = 0.003), rs4402960 (b = −0.057, p = 0.046) and rs10830963 (b = −0.096, p = 0.001) were associated with HOMA-B, while rs7754840 was associated with decrease in insulin AUC during a 100 g OGTT given at the time of GDM diagnosis (b = −0.080, p = 0.007).

**Conclusions/Significance:**

Several risk alleles of type 2 diabetes were associated with GDM in pregnant Chinese women. The effects of these SNPs on GDM might be through the impairment of beta cell function and these risk loci contributed additively to the disease.

## Introduction

Gestational diabetes mellitus (GDM) is defined as glucose intolerance with onset or first recognition during pregnancy [1]. It affects 5% to 10% of Asian women with an increasing trend observed in some developing countries, including China [2]. Epidemiological studies have confirmed that GDM is associated with increased feto-maternal morbidity and long-term complications in mothers and offsprings. However, the pathogenesis of GDM is still largely unknown. Given the fact that women with a history of GDM are at an increased risk of developing type 2 diabetes (T2D) later in their lives [3] and women with a family history of diabetes may be predisposed to an increased risk of GDM [4], GDM may share the same risk factors and genetic susceptibilities with T2D.

Genome-wide association studies (GWAS) and large-scale genetic linkage analyses about GDM have not been reported lately. So the strategy used to identify genes which underlie the genetic causes of GDM is mainly through a candidate gene approach and the majority of the identified candidate genes for GDM came from those associated with T2D. Recently, spectacular advance in identifying susceptible genes involved in T2D in the Western population has been made by using GWAS [5]–[9]. The effects of some candidate genes were replicated in Chinese populations [10]–[17] and these genes mainly included cyclin-dependent kinase 5 regulatory subunit associated protein 1-like 1 (*CDKAL1*), insulin-like growth factor 2 mRNA binding protein 2 (*IGF2BP2*), gene regions of cyclin-dependent kinase inhibitor 2A and 2B (*CDKN2A-CDKN2B*), solute carrier family 30 (zinc transporter), member 8 (*SLC30A8*), potassium voltage-gated channel, KQT-like subfamily, member 1 (*KCNQ1*), and peroxisome proliferator-activated receptor gamma (*PPARG*). In the meanwhile, the GWAS on Chinese population discovered two new genes, protein tyrosine phosphatase, receptor type, D (*PTPRD*) and serine racemase (*SRR*), as the candidate genes of T2D [16]. Positive association between GDM and some of the new diabetogenic genes was also observed [18]–[25].

It has been reported that the pathophysiological changes of GDM are similar to those observed in T2D, which is characterized by peripheral insulin resistance accompanied by an insulin secretory defect [26]–[28]. Functional studies showed that these new diabetogenic genes took part in many steps of the process, for instance, impaired beta cell function (*CDKAL1, SLC30A8, CDKN2A/B, IGF2BP2, KCNQ1, MTNR1B*), insulin resistance (*PPARG*), and abnormal utilization of glucose (*GCK*) [24], [29]–[36].

Considering the lack of sufficient evidence about the effect of candidate genes of T2D on GDM and the discrepancy among races [2], [37], we aimed to determine whether the results derived from other races are applicable to the Chinese population, or whether different genetic background can cause different quantitative traits related to GDM. Moreover, the replication performed in multiple ethnicities did help to identify population specific risk variants. In the study, we selected six genes, including *CDKAL1*, *IGF2BP2*, *MTNR1B*, *GCK*, *SRR* and *CDKN2A-CDKN2B*, and tested their association with GDM in Chinese pregnant women. The results may provide additional insights to the mechanisms which underlie the genetic variants associated with the risk of GDM.

## Methods

### Ethnic statement

Written informed consent was obtained from each participants, and the study was approved by the Institutional Review Board of Peking Union Medical College Hospital.

### Study subjects

During the period of 2006 to 2010, we recruited a total of 1,764 participants of Chinese pregnant women residing in Beijing from Peking Union Medical College Hospital. The pregnant women without a previous diagnosis of glucose intolerance were routinely screened for GDM between 24 and 28 weeks of gestation by two procedures. First of all, a 50 g glucose challenge test (GCT) was used as preliminary screening. It was considered as GCT negative (GCT−) if the plasma glucose concentration was less than 7.8 mmol/l after glucose intake 1 hour. Otherwise, diagnosed as GCT positive (GCT+). The pregnant women of GCT+ were then given a 100 g oral glucose tolerance test (OGTT). Diagnosis of GDM was based on the criteria as set by the American Diabetes Association [38]. The glucose threshold values were as follow: fasting 5.3 mmol/l, 1 h 10.0 mmol/l, 2 h 8.6 mmol/l and 3 h 7.8 mmol/l. A diagnosis of GDM was made if 2 or more of the glucose values met or exceeded the threshold value. Normal glucose tolerance (NGT) was diagnosed when all plasma glucose values were below the threshold values. Based on the above criteria, 725 subjects with GDM, 641 with NGT, and 275 GCT− participants were recruited to the study. The NGT and GCT− groups were taken as controls.

### Clinical and biochemical data

Clinical and biochemical data of all subjects were collected at 24–28 weeks gestation. Clinical data included age, height, weight at one year before pregnancy, systolic blood pressure and diastolic blood pressure. The family history of T2D in each subject was also recorded. Body mass index before gestation (pre-BMI) was calculated as body weight (kg) divided by the square of height (m^2^).

Biochemical data consisted of fasting plasma glucose (FPG), fasting plasma insulin (FPI), glycated hemoglobin, serum triacylglycerol, total cholesterol, HDL-cholesterol, LDL-cholesterol, high sensitivity C-reactive protein, white blood cell and platelet counts.

Homeostatic model assessment (HOMA) data and the area under the curve (AUC) of insulin during a 100 g OGTT performed at the time of GDM diagnosis were calculated to assess insulin resistance and beta-cell function. Homeostasis model assessment of insulin resistance (HOMA-IR) was calculated by (FPI in mU/L×FPG in mmol/l)/22.5. Homeostasis model assessment of beta-cell function (HOMA-B) was calculated by (FPI in mU/L×20)/(FPG in mmol/l−3.5) as previously reported [39]. Another assessment index of beta-cell function, the AUC of insulin at 3 hour was evaluated according to the trapezoid method: V1+V2+0.5*V0+0.5*V3, where V is the insulin concentration at the indicated time [40].

### SNP selection, Genotyping and Genotype quality control

The loci previously reported to be associated with type 2 diabetes at a genome-wide significance level were selected, including *IGF2BP2* (rs4402960), *SRR* (rs391300), *MTNR1B* (rs10830963) and *CDKAL1* (rs7754840). Another two representative SNPs (rs2383208 in CDKN2A–CDKN2B and rs4607517 in GCK) that were in the strong linkage disequilibrium with rs10811661 (D′ = 0.931, r^2^ = 0.847) and rs1799884 (D′ = 0.961, r^2^ = 0.924), respectively, were also selected [24], [41]. Genotyping was performed using Taqman allelic discrimination assays. The quality value was set as 95% during data analysis using the Sequence Detection System version 2.4 software (Applied Biosystems). Genotyping quality control was performed in 10% of the samples by duplicate checking (rate of concordance in duplicates >99%). Genotyping success rate was similar for women with gestational diabetes mellitus and for control subjects. The genotyping call success rates were 98.1%, 98.5%, 97.7%, 97.3%, 94.6% and 98.0% for rs4402960, rs2383208, rs4607517, rs7754840, rs391300 and rs10830963, respectively.

### Statistical analysis

The quantitative variable with normal distribution (platelet count) was given as mean ± standard deviation (SD), and quantitative variables with non-normal distribution were given as medians and interquartile range. The continuous data (HOMA-B, HOMA-IR, AUC of insulin, total cholesterol, triacylglycerol, and HDL-cholesterol) were log-transformed to approximate normal distributions. Quantitative data with normal distribution or log-transformed variables were analyzed by student's *t* test. Nonparametric tests were performed to analyze the other variables.

The chi-square tests were used to determine whether individual polymorphism was in Hardy–Weinberg equilibrium. Genotypes were given codes of 0, 1 and 2, and the odds ratio (OR) was expressed per difference in the number of risk alleles. A multiple logistic regression model was used to investigate the individual effect of these genes on GDM. These analyses were based on additive, recessive and dominant models, and adjusted for age and the family history of type 2 diabetes. The ORs with 95% confidence intervals (CIs) were presented. Multiple linear regression models with adjustment for age were also applied to analyze these quantitative traits, and the regression coefficients (b) were presented. A two-sided *p* value <0.05 was considered statistically significant. The statistical analyses were performed using SPSS 11.0 (SPSS Inc, Chicago, IL, USA).

The following assumptions were made for the power calculation: a prevalence of GDM equal to 3%, a high-risk allele frequency of 0.20, and an effect size of 1.3. By studying a sample of 725 cases and 1039 controls, our present study had more than 80% power, under a multiplicative model, with a type I error rate of 0.05. When the predisposing allele frequency was >30%, the study had at least 80% power to detect an OR of 1.22 under a multiplicative model. Power calculations were performed using the Genetic Power Calculator, available at http://ibgwww.colorado.edu/~pshaun/gpc/.

## Results

### Clinical and biochemical parameters

The clinical and biochemical parameters of the control and GDM groups were presented in Table 1. Mean age, systolic and diastolic blood pressure, FPG, FPI, glycated hemoglobin protein, serum triacylglycerol, high sensitivity C-reactive protein, white blood cell and platelet counts were significantly higher in the GDM group than controls (*p*<0.001), whereas pre-BMI was similar in both groups (*p* = 0.086). In addition, women with GDM displayed higher HOMA-IR, lower HOMA-B and higher AUC of insulin (*p*<0.001).

**Table 1:** Clinical characteristics of the study participants.

	Controls (n = 1039)	GDM (n = 725)	P value
Age (years)	30.00 (28.00, 33.00)	32.00 (30.00, 35.00)	<0.001
Pre-BMI (kg/m2)	21.48 (19.57, 23.62)	21.72 (19.89, 24.04)	0.086
Systolic blood pressure (mmHg)	110.00 (102.00, 120.00)	114.00 (107.00,123.00)	<0.001
Diastolic blood pressure (mmHg)	67.00 (61.00, 73.00)	70.00 (63.00, 76.00)	<0.001
Fasting plasma glucose (mmol/l)	4.50 (4.30, 4.70)	4.80 (4.50, 5.20)	<0.001
Fasting plasma insulin (mU/l)	6.10 (4.33, 8.90)	7.60 (5.20, 11.30)	<0.001
Glycated hemoglobin protein (%)	5.20 (5.00, 5.30)	5.40 (5.20, 5.70)	<0.001
HOMA-B	131.43 (91.67, 191.11)	115.56 (81.13, 177.05)	0.019
HOMA-IR	1.20 (0.84, 1.80)	1.64 (1.08, 2.59)	<0.001
AUC of insulin during 100 g OGTT at the time of diagnosis of GDM (mU l^−1^×h)	156.28 (108.28,218.50)	198.90 (141.38,285.51)	<0.001
White blood cell count (*10^12^/l)	8.86 (7.62, 10.2)	9.40 (8.14, 10.82)	<0.001
Platelet count (*10^9^/l)	225.70±50.21	240.91±53.51	<0.001
High sensitivity C-reactive protein (mg/l)	2.19 (1.33, 4.36)	3.24 (1.75, 5.80)	<0.001
Total cholesterol (mmol/l)	6.10 (5.43, 6.74)	6.06 (5.29, 6.72)	<0.001
Triacylglycerol (mmol/l)	2.21 (1.81, 2.74)	2.54 (2.01, 3.19)	<0.001
HDL-cholesterol (mmol/l)	2.10 (1.81, 2.38)	1.99 (1.73, 2.28)	<0.001
LDL-cholesterol (mmol/l)	3.37 (2.79, 3.94)	3.25 (2.68, 3.87)	0.007

Platelet count was the quantitative variable with normal distribution and was given as means ± standard deviation.

Data was given as medians (interquartile range) for the quantitative variables with non-normal distribution.

Seven variables (platelet count, HOMA-B, HOMA-IR, AUC of insulin, total cholesterol, triacylglycerol and HDL-cholesterol) were log-transformed to approximate normal distributions and were analyzed by student's *t* test. The other variables in table 1 were analyzed using the nonparametric tests.

### Genotype and allele analysis

All single nucleotide polymorphisms were in Hardy–Weinberg equilibrium. We first examined the potential effects of the six different SNPs on GDM susceptibility in our Chinese case-control samples. The results were shown in Table 2. We discovered that GDM was associated with rs2383208 (OR = 1.242, 95% CI = 1.077–1.432, p = 0.003), rs4402960 (OR = 1.207, 95% CI = 1.029–1.417, p = 0.021) and rs391300 (OR = 1.202, 95% CI = 1.020–1.416, p = 0.028). Compared with wild-type carriers, homozygous harboring the risk alleles of rs4402960, rs2383208 and rs391300 had a 1.498-fold (95%CI = 1.002–2.240, p = 0.049), a 1.532-fold (95% CI = 1.140–2.060, p = 0.005) and a 1.856-fold (95%CI = 1.236–2.789, p = 0.003) increased risk of gestational diabetes mellitus, respectively. In addition, the effect size was greater under a recessive model in rs391300 (OR = 1.802, 95%CI = 1.226–2.701, p = 0.003) and it changed slightly under a dominant model in rs4402960 (OR = 1.232, 95%CI = 1.008–1.507, p = 0.042). The relation between other SNPs (rs7754840, rs10830963 and rs4607517) and GDM was not observed.

**Table 2:** Genotype and allele distributions and corresponding odds ratios for gestational diabetes mellitus.

SNP (Gene)	Genotype or risk allele	GDM Number (%)	Controls Number (%)	Additive model p value, and OR (95% CI)	Dominant model p value and OR (95% CI)	Recessive model p value and OR (95% CI)
rs4402960 (*IGF2BP2*)	TT	56 (7.9)	59 (5.8)	**0.049**; 1.498 (1.002–2.240)	**0.042** 1.232(1.008–1.507)	0.095 0.398(0.943–2.072)
	GT	278 (39.4)	361 (35.2)	0.108; 1.189 (0.963–1.469)		
	GG	371 (52.6)	605 (59.0)	1		
	T	390 (27.7)	479 (23.4)	**0.021**; 1.207(1.029–1.417)		
rs2383208 (*CDKN2A2B*)	AA	280(39.1)	330 (32.3)	**0.005**; 1.532 (1.140–2.060)	**0.008** 0.754(0.613–0.928)	**0.031** 0.744(0.569–0.973)
	AG	328(45.7)	497 (48.7)	0.177; 1.217 (0.915–1.619)		
	GG	109(15.2)	194 (19.0)	1		
	A	888(61.9)	1157(56.7)	**0.003**; 1.242(1.077–1.432)		
rs391300 (*SRR*)	TT	58 (8.8)	55 (5.5)	**0.003**; 1.856(1.236–2.789)	0.229 1.133(0.924–1.389)	**0.003** 1.820(1.226–2.701)
	CT	283 (42.7)	431 (42.8)	1.044; 0.689(0.844–1.292)		
	CC	321(48.5)	520 (51.7)	1		
	T	399 (30.1)	541 (26.9)	**0.028**; 1.202(1.020–1.416)		
rs10830963 (*MTNR1B*)	GG	137(19.6)	191 (18.6)	0.189; 1.215(0.909–1.626)	0.119 1.190(0.956–1.481)	0.476 1.096(0.852–1.411)
	CG	364(52.0)	509 (49.5)	0.159; 1.180(0.937–1.478)		
	CC	199(28.4)	329 (32.0)	1		
	G	638(45.6)	891(43.3)	0.152; 1.111 (0.962–1.282)		
rs4607517 (*GCK*)	AA	37 (5.3)	49 (4.8)	0.602; 1.131(0.713–1.793)	0.850 1.020(0.832–1.251)	0.602 1.129(0.717–1.778)
	AG	244(34.8)	356 (34.8)	0.965; 1.005(0.812–1.243)		
	GG	1420(59.9)	618 (60.4)	1		
	A	318(22.7)	454(22.2)	0.726; 1.031(0.870–1.221)		
rs7754840 (*CDKAL1*)	CC	159(22.8)	197 (19.3)	0.097; 1.274(0.957–1.695)	0.518 1.075(0.863–1.340)	0.055 1.273(0.995–1.627)
	CG	339(48.6)	512 (50.2)	0.991; 1.001(0.793–1.695)		
	GG	199(28.6)	311 (30.5)	1		
	C	657(47.1)	906(44.4)	0.127; 1.117(0.969–1.289)		

P values<0.05 were shown in bold; P values were adjusted for age and family history of type 2 diabetes (T2D) using the logistic regression analysis, but not corrected for multiple comparisons.

Subsequently we tested the joint effects of risk alleles of susceptible loci on GDM to investigate if these loci affected the disease additively. Here we just selected SNPs with p values less than 0.05 (rs4402960, rs2383208 and rs391300) and calculated the joint effects by summing up the number of risk alleles for each participant who had the genotyping information of all these three SNPs. We found that the proportion of women with GDM increased in the subgroups with more risk alleles, the subgroups carrying more risk alleles had a significantly higher risk for GDM, with each additional risk allele increased GDM risk by 1.196-fold (95%CI = 1.092–1.309, P = 1.08×10^−4^). Moreover, the subjects who harbor 4, 5 and 6 risk alleles have a 2.008-fold (p = 0.011), 5.576-fold (p = 3.31×10^−4^) and 9.717-fold (p = 0.047) increasing in the odds of developing GDM as compared to individuals without any risk alleles, respectively. All the analysis was based on the adjustment for age and the family history of T2D.

### FPG, HOMA-B, HOMA-IR and AUC of insulin

We analyzed the association between each SNP and quantitative traits in the research (as shown in table 3). The risk allele of rs10830963, rs2383208 and rs391300 showed association with increased FPG (p = 0.019, p = 0.034, p = 0.028, respectively). We further observed that these variants exerted combined effects on FPG, with a mean 0.087 mmol/L increase per risk allele (95%CI = 0.011–0.084, p = 0.012).

**Table 3:** Associations between risk alleles and FPG, insulin beta cell function and insulin resistance.

SNP	Effect allele*/other allele		FPG (mmol/L)	HOMA-B	AUC of insulin during 100 g OGTT at the time of diagnosis of GDM (mU h×L^−1^)	HOMA-IR
rs4402960	T/G	b	0.033	−0.057	−0.006	−0.027
		95%CI	−0.017∼0.076	−22.787∼−0.211	−11.175∼8.940	−0.212∼0.074
		P	0.214	**0.046**	0.828	0.345
rs2383208	A/G	b	0.055	−0.085	−0.005	0.005
		95%CI	0.003∼0.085	−58.160∼−12.101	−9.597∼7.935	−0.113∼0.135
		P	**0.034**	0.003*	0.852	0.862
rs391300	T/C	b	0.059	0.002	0.020	0.017
		95%CI	0.006∼0.100	−13.872∼15.133	−6.791∼13.633	−0.101∼0.188
		P	**0.028**	0.085	0.511	0.555
rs10830963	G/C	b	0.062	−0.096	−0.016	−0.020
		95%CI	0.008∼0.091	−33.989∼−9.013	−11.509∼6.485	−0.170∼0.079
		P	**0.019**	**0.001**	0.584	0.476
rs4607517	A/G	b	0.040	0.010	0.007	0.017
		95%CI	−0.011∼0.087	−12.130∼17.193	−9.216∼11.916	−0.104∼0.194
		P	0.127	0.735	0.802	0.554
rs7754840	C/G	b	0.046	−0.019	−0.080	0.000
		95%CI	−0.005∼0.078	−16.663∼8.285	−20.836∼−3.337	−0.126∼0.126
		P	0.081	0.510	**0.007**	0.997

P values<0.05 were shown in bold. P values were adjusted for age but not corrected for multiple comparisons.

Log transformed (log10) values were used for HOMA-B, HOMA-IR and AUC of insulin during 100 g OGTT at the time of diagnosis of GDM.

We also obtained homeostatic model assessment data for beta-cell function and insulin resistance (HOMA-B and HOMA-IR, respectively) in both groups. The loci, rs4402960 and rs10830963, were statistically associated with HOMA-B (p = 0.046, p = 0.001, respectively). For rs2383208, we found that carriers of genotype-AA showed lower level of HOMA-B (p = 0.002, the result was not listed in table 3). The significance remained in the recessive model using the logistic regression analysis (p = 0.003). Further analyses of the risk alleles (A-allele of rs4402960, A-allele of rs2383208 and G-allele of rs10830963) confirmed their joint effects on HOMA-B level (b = −0.108 unit per risk allele, 95%CI = −20.922∼−6.546, p = 1.86×10^−4^). We just discovered that rs7754840 was significantly correlated with AUC of insulin (b = −0.080 mU l^−1^×h per risk allele, 95%CI = −20.836∼−3.337, p = 0.007). For HOMA-IR, no significant association was detected.

## Discussion

In the present study, we observed that some common variants conferring susceptibility to type 2 diabetes mellitus may increase the risk of GDM in pregnant Chinese women. The results also confirmed that the polymorphism in *SRR* was associated with GDM in the Chinese population for the first time.

### *IGF2BP2* rs4402960

Our results provided evidence that rs4402960 was a susceptible gene locus for GDM in Chinese pregnant women (OR = 1.207, 95% CI = 1.029–1.417, p = 0.021). This result was similar to that observed by a Korean GDM study (OR = 1.18, 95%CI = 1.01–1.38, p = 0.034) [23], but differed from that of the Danish (OR = 1.18, 95%CI = 0.97–1.42, p = 0.096) [24]. This discrepancy might be attributed to racial differences [2], [37]. A recent study have showed an association between rs4402960 and T2DM in a case-control sample living in Beijing (OR = 1.19, 95%CI = 1.04–1.37, p = 0.009) [15]. The results of our study showed a similar effect which further suggested that GDM may share the similar genetic background with T2D.

It has been reported that the variants of *IGF2BP2* can affect first-phase insulin secretion and the disposition index [29]. In our research we found that the subjects harboring the risk T allele of rs4402960 showed a negative association with HOMA-B (b = −0.057, p = 0.046) but not with HOMA-IR and fasting glucose level. The results indicated that common variation in *IGF2BP2* mainly affected beta cell function rather than insulin sensitivity or fasting glucose level. It confirmed a previous study in women with GDM and in accordant with the dominant role of beta cell dysfunction in GDM [42], [43].

### *CDKN2A-CDKN2B* rs2383208

We found that rs2383208, a variant at the same LD block with rs10811661 [24], was a risk locus for GDM in Chinese population (OR = 1.242, 95% CI = 1.077–1.432, p = 0.003). The finding was consistent with the previous study on Korean GDM women [23], though the OR in our study was a little lower than that in their research. On the other hand, a lack of association at rs10811661 in Danish women with previous GDM was observed, partly due to a lower effect size (OR = 1.12, 95%CI = 0.87–1.45, p = 0.39) [24].

Because the relation between *CDKN2A-2B* and beta cell function has been widely reported in Western and Eastern populations [30], [44], [45], we also determined the association between rs2383208 and beta cell function. However, we only found that carriers of rs2383208 allele-A showed lower levels of HOMA-B, and lack association with AUC of insulin. The discrepancy may be largely caused by differences between the two indexes. The directly measured insulin data rather than surrogate measures HOMA-B and AUC of insulin may improve the specificity. In addition, our finding that rs2383208 was associated with FPG and HOMA-B, to some content, support the view that impaired beta cell function and hyperglycemia likely share the same underlying pathogenic mechanism [33], [41]. Therefore, the risk allele of rs2383208 may be associated with an increased risk of GDM primarily by regulating the secretion of pancreatic beta cell and FPG.

### *SRR* rs391300

*SRR* rs391300 was originally identified as genetic determinants of type 2 diabetes by GWA studies on Han Chinese in 2009 [16]. In our study, we detected its association with GDM in Chinese population and found a nominal role of this variant on the risk of GDM (OR = 1.202, 95%CI = 1.020–1.416, p = 0.028). In addition, we discovered that risk allele-T of rs391300 showed association with FPG (b = 0.059 mmol/L per allele, p = 0.028) but not with beta cell function or insulin resistance. All these findings indicated that SRR variant may affected the incidence of GDM by modulating the secretion of insulin and/or glucagon as reported previously [16].

### *MTNR1B* rs10830963

We observed that rs10830963 was not associated with GDM in Chinese women. However, we found that this variant showed moderate association with HOMA-B (b = −0.096, p = 0.001) and FPG (b = 0.062, p = 0.019). Previous studies indicated that the *MTNR1B* variants were significantly associated with increased fraction of glycated hemoglobin and reduced beta-cell function (HOMA-B), and not related to fasting insulin level or insulin sensitivity [46], [47]. One possible explanation was that *MTNR1B* may down regulate GCK expression and glucose-stimulated insulin secretion by lowering intracellular cAMP level [33], [48], [49]. Another study also demonstrated that rs10830963 was associated with GDM by affecting islet beta cell function and fasting glucose level [25]. In our study, carriers of the risk allele G of rs10830963 showed a lower value of HOMA-B and higher level of FPG. Our finding in the subpopulation further emphasized the importance of rs10830963 for beta-cell function and FPG.

### *GCK* rs4607517

A study in Scandinavian women showed rs1799884 in *GCK* was a candidate locus for GDM [22]. We did not find the similar association between rs4607517 and GDM in our study though the rs4607517 and rs1799884 exhibited strong linkage disequilibrium [41]. Furthermore, we did not find the correlation between rs4607517 and quantitative traits (FPG and HOMA-B) as reported previously [33], [41], [47]. One possible explanation for this contrasting result may be attributed to how much *GCK* rs4607517 affected these traits. In our study, the effect size of rs4607517 on FPG (0.04 mmol/l) was a little smaller than that of the other three SNPs (0.055–0.062 mmol/l FPG per allele) and than that of rs4607517 in previous study (0.06 mmol/l per allele) [47].

### *CDKAL1* rs7754840

*CDKAL1* was originally recognized as a candidate gene for T2DM by several GWAS [6], [7], [10], [11], [13]. The rs7754840 in *CDKAL1* was associated with T2DM in the Chinese population (OR = 1.127, 95%CI = 1.027–1.238, P = 0.0119) [17]. Recent studies have indicated that the variation in *CDKAL1* involved in the pathogenesis of GDM with an OR range from 1.22 to 1.55 [23], [24]. Unfortunately, we did not find the relation between rs7754840 and GDM in our study. A relatively lower effect (OR = 1.117, 95%CI = 0.969–1.289, p = 0.127) might result in this inconsistent conclusion. Previous study indicated that CDKAL1 probably plays a role in the regulation of insulin secretion, even under glucotoxic conditions [24], [35], [36]. In the study, we found that rs7754840 showed significant association with insulin AUC (b = −0.080 mU l^−1^×h, p = 0.007), which was consistent with a Korean study [23] and further indicated the role of CDKAL1 variants on beta cell function.

### Combined genetic risk of GDM

Individuals carrying more risk alleles had a higher risk of type 2 diabetes [17], [34]. This additive effect of the variants on GDM with an OR of 1.18 per risk allele (95% CI = 1.10–1.27, *P* = 3.2×10^−6^) was also observed by Lauenborg [24]. In our study, subjects who harbor more than 4 risk alleles have at least a 2.008-fold increase for developing GDM as compared with individuals who did not carry any risk alleles. Similarly, the combined effects of the SNPs on HOMA-B and FPG were much obvious in contrast to the effects of single SNP as described in our research. These results support the finding of an additive effect of the type 2 diabetes risk alleles on the risk for GDM.

There are some limitations in the present study. First, although the study included 725 women with GDM and 1039 controls, the statistical power of the sample was not large enough to detect a weak effect size (OR<1.2). As a result, some associations may have been overlooked. Second, it was not confirmed whether all of the subjects in the control group had experienced pregnancy without GDM. In our study population, there were 271 GCT (−) women who were not given the 100 g OGTT after a 50 g glucose challenge test. However, the effects of this overlook on our interpretation of the results should be minimal because the prevalence of GDM in GCT (−) pregnant women was estimated to be very low [50].

Our study demonstrated that several previously proven type 2 diabetes risk alleles were associated with GDM in pregnant Chinese women. The study also provided evidence of the strong genetic background for the development of GDM in a multigenetic manner. Compared to women who did not harbor any risk allele, women carrying at least five or more risk alleles had a higher risk of developing GDM. The effects of these SNPs on GDM may be through the impairment of beta cell function. Further studies are required to assess the relationship between these polymorphisms and GDM in other ethnicities.
